# Association Between the Japanese Version of the Stanford Integrated Psychosocial Assessment for Transplantation and Adjustment Disorder in Japanese Patients Using Ventricular Assist Devices as a Bridge to Heart Transplantation

**DOI:** 10.7759/cureus.73828

**Published:** 2024-11-16

**Authors:** Junko Tsutsui, Hidehiro Oshibuchi, Sayaka Kobayashi, Motoharu Yamanaka, Natsumi Endo, Yuki Ichihara, Rie Akaho, Katsuji Nishimura

**Affiliations:** 1 Department of Psychiatry, Tokyo Women's Medical University, Tokyo, JPN; 2 Department of Psychiatry, Saitama Medical Center, Saitama Medical University, Saitama, JPN; 3 Adult Nursing, Japanese Red Cross College of Nursing, Tokyo, JPN; 4 Department of Nursing, Tokyo Women’s Medical University, Tokyo, JPN; 5 Department of Cardiovascular Surgery, Tokyo Women's Medical University, Tokyo, JPN

**Keywords:** adjustment disorder, delirium, psychiatric disorder, stanford integrated psychosocial assessment for transplantation, ventricular assisting device

## Abstract

Objective: Ventricular assist device (VAD) serves as either a bridge to transplantation (BTT) or destination therapy (DT) for end-stage heart failure. In Japan, the extended wait time for heart transplants can make VAD usage for BTT comparable in duration to DT in other countries. Previous studies suggest that while DT patients experience improved quality of life post-VAD implantation, BTT patients often see a decline after two years. This study aims to explore the association between scores of the Japanese version of the Stanford Integrated Psychosocial Assessment for Transplantation (SIPAT-J), adverse medical events (AMEs) and psychiatric disorders, particularly adjustment disorder (AD), in Japanese BTT patients.

Methods: A retrospective analysis was conducted on 24 Japanese patients who underwent VAD implantation for BTT between August 2014 and December 2019. The SIPAT-J, which evaluates the patient’s readiness level, social support system, psychological stability and psychopathology, and lifestyle and effects of substance use, was applied to medical records. Psychiatric diagnoses were reviewed to identify AMEs, AD and other conditions. Patients were grouped by AMEs and AD status, and SIPAT-J scores were dichotomized at the median to examine potential associations. Statistical analysis was performed using Fisher's Exact test.

Results: There were no significant associations found between SIPAT-J scores and the development of AD or AMEs, likely due to the small sample size and varied observation periods, and differences in timing between diagnosis, evaluation, and VAD implantation. However, more than half of the patients developed psychiatric symptoms after VAD implantation, with delirium and insomnia being the most common. Identified stressors for AD included deteriorating family relationships, difficulty accepting heart disease, and future uncertainty. AMEs, which have been linked to AD onset in previous research, were also present in several cases.

Discussion: This study is the first to examine the relationship between any version of SIPAT and psychiatric outcomes in BTT patients. The results suggest that while SIPAT-J captures important psychosocial factors, additional factors related to VAD-induced lifestyle changes and family dynamics may be crucial for predicting AD risk. Therefore, future research should consider a more detailed psychosocial evaluation to capture patient expectations about post-VAD life. Although preliminary, this study underscores the need for comprehensive psychosocial screening in BTT patients to facilitate early intervention and support for those at risk of AD.

## Introduction

Ventricular assist devices (VAD) are mechanical devices that partly or completely replace the function of the left ventricle in patients with end-stage heart failure [1]. VAD can be used either as a bridge to transplantation (BTT), which provides temporary support or as destination therapy (DT), which supports patients for their lifetimes [2].

 In Japan, the waiting time for patients with BTT to undergo heart transplantation is much longer than in other countries because of donor organ shortage [3]. Therefore, the duration of living with a VAD may be similar in Japanese BTT patients and those in other countries receiving VAD support as DT [4]. Previous research has shown that, whereas DT patients’ quality of life improves significantly from pre-implantation to one year after VAD implantation and remains stable thereafter, it declines to pre-implantation levels two years after VAD implantation in BTT patients [5]. In another study, half of the participants with BTT developed adjustment disorder (AD)[6]. A possible explanation for this apparent discrepancy between DT and BTT is that some BTT patients become increasingly concerned about their eligibility for transplantation as the waiting period for heart transplantation increases, especially given the potential for severe complications [5]. This prolonged distress can trigger AD. Therefore, it is important to identify individuals who are psychosocially vulnerable and at risk of developing psychosocial distress such as AD after VAD implantation and to start supporting them at an early stage.

Today, the most widely used tool for psychosocial assessment of organ transplantation candidates, including VAD patients, is the Stanford Integrated Psychosocial Assessment for Transplantation (SIPAT) [7,8]. In several studies, SIPAT scores have been shown to be associated with adverse medical events (AMEs) after VAD implantation [9,10]. However, SIPAT scores based on data recorded prior to VAD implantation and their association with psychosocial disorders, such as AD, have not yet been investigated in individuals with VAD as BTT whose conditions have been diagnosed precisely by psychiatrists. Therefore, in the present study, we aimed to investigate associations between scores on the Japanese version of SIPAT (SIPAT-J), the presence of AMEs and psychosocial disorders, especially AD, diagnosed by psychiatrists after VAD implantation in Japanese BTT patients [11].

This article was previously posted to the Research Square preprint server on 25 September 2023.

## Materials and methods

Data collection

This retrospective, single-center, opt-out study was conducted at an urban university hospital in Japan. Two of the 26 patients who had undergone VAD implantation from 1 August 2014 to 31 December 2019 were excluded, one who was younger than 16 and one who had been evaluated at another institute and for whom we had insufficient information for inclusion in the present study. The medical records of the remaining 24 patients were reviewed starting from the day of psychiatric evaluation until 31 December 2020.

Psychiatric evaluation was mandatory for VAD implantation. Although SIPAT-J had not yet been developed, the translation process was underway, and the evaluation covered the items included in SIPAT. Data collection was censored at the time of death or heart transplantation. The study was approved by the Ethics Committee on Human Research of the Institutional Review Board of Tokyo Women’s Medical University (Approval No. 5779).

Japanese version of the Stanford Integrated Psychosocial Assessment for Transplantation (SIPAT-J)

SIPAT-J consists of 18 items classified into four domains, as does the original version [11]. The domains are patient’s readiness level, social support system, psychological stability and psychopathology, and lifestyle and effects of substance use. The SIPAT-J was retrospectively applied to the medical records of the identified patients. The electronic medical records of each participant were printed out and provided to the rater, who is an expert at evaluating transplantation candidates (SK), including only the sections relevant to the medical data, psychiatric evaluation interviews, and the transplant coordinator’s notes at the time prior to VAD implantation but not physical or psychological outcomes after VAD implantation. SK also served as one of the raters in a previous retrospective SIPAT-J study, which demonstrated excellent inter-rater reliability (Pearson's correlation coefficients: 0.82-0.90) [11]. The SIPAT-J scoring was conducted with the evaluation criteria used in this prior study. After evaluation, the total and four domain scores of SIPAT-J were calculated. The cut-off point for the total score on the original SIPAT is known to be 21 [8]. However, the cut-off point for SIPAT-J is unknown. Therefore, we considered that patients with SIPAT-J scores greater than the median of the present data were at greater psychosocial risk and that those with lower scores than the median were at lower risk.

Psychiatric diagnosis and AMEs after VAD implantation

The first author (JT) reviewed the following: medical records by the primary attending physician during the study period, especially the summary records made at the end of the year, to investigate AMEs. AMEs included driveline infection, gastrointestinal bleeding, pump thrombosis and stroke, and psychiatrists' and clinical psychologists' records during the study period to investigate the prevalence of psychiatric disorders during VAD implantation. The date of diagnosis and corresponding information were collected for patients in whom psychiatric disorders had been diagnosed. Psychiatric diagnoses were made by a psychiatrist in accordance with the ICD 10 classification.

Data analysis

The primary aim of this study was to investigate the association between SIPAT-J scores and the development of AD after VAD implantation. AMEs were considered to be a major trigger of AD; therefore, participants were classified based on whether they developed AD, whether AMEs occurred prior to AD, and whether their SIPAT-J scores (total score and the four domains) were above the median of the study population or not. The data were entered into a 4x2 table. The Fisher’s Exact test was conducted to examine the differences between groups. Statistical analyses were performed by JMP (Version 16.0, SAS Institute, Cary, NC, USA; 1989-2021). p<0.05 was accepted as denoting statistical significance.

## Results

Clinical characteristics

The medical records of 18 male (75%) and 6 female (25%) participants were reviewed. The average age at the time of the psychosocial assessment was 42.0 (SD±11.7) years, and 18 (75%) of the participants had dilated cardiomyopathy. All patients had VAD implanted for BTT purposes.

AMEs prior to adjustment disorder

 During the study period, 11 AMEs occurred in 10 (45.5%) participants, including 8(72.7%) strokes, 2 (18.2%) driveline infections and １(9.1%) pump thrombosis in total. No patient had gastrointestinal bleeding.

Psychiatric diagnoses

**Figure 1:**
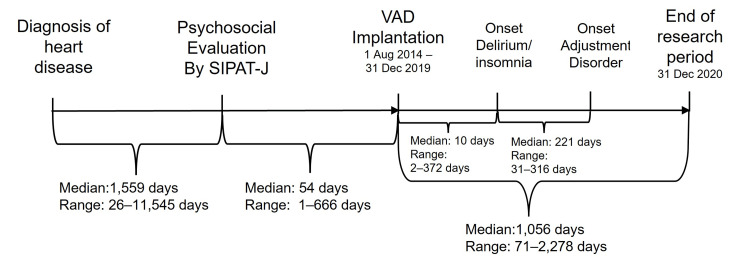
Duration of each event starting from the diagnosis of heart disease until the end of research SIPAT-J: Japanese version of Stanford Integrated Psychosocial Assessment for Transplantation, VAD: Ventricular assist device.

Within 10 days (median) after the VAD implantation, 14 patients (58%) were referred to the psychiatry team. No included patients were assessed outside the institution. The breakdown of diagnoses was delirium and/or insomnia (12 patients), AD (5 patients), remission of depression (one patient) and higher brain dysfunction (one patient). There was an overlap in diagnoses because the same patient was referred several times. Of these patients, all patients with a diagnosis of AD, which is the focus of this study, had once been diagnosed with delirium and/or insomnia after VAD was implanted. The duration of each event, starting from the diagnosis of heart disease until the end of research, is shown in Figure 1.

Identified stressors for AD were deterioration of relationships with family members, irritation about persistent insomnia, difficulty in accepting the reality of having serious heart disease, and worries about the future. Some patients had multiple stressors.

Association between AMEs, AD and SIPAT-J scores

Table 1 shows the number and percentage of participants in 8 groups divided by the presence of AMEs and AD and whether their SIPAT-J scores were lower or higher than the median scores. Because the focus of the present study was to examine the association between SIPAT-J scores and the development of AD after VAD implantation, patients diagnosed with remission of depression and high brain dysfunction were excluded. Fischer’s Exact test was performed to investigate the association between the groups. However, no association was detected (p=0.22-1.00).

**Table 1:** SIPAT-J scores by onset of adjustment disorder after implantation of VAD and adverse medical events prior to adjustment disorder SIPAT-J: Japanese version of Stanford Integrated Psychosocial Assessment for Transplantation, VAD: ventricular assist device, AMEs: adverse medical events. *The occurrence of more than one of the following events was defined as AMEs positive: Driveline infection, pump thrombosis, gastrointestinal bleeding, pump thrombosis or stroke. **One patient diagnosed as in remission of depression and one diagnosed as having higher brain dysfunction were excluded.

SIPAT-J domains	Scores	No AMEs* nor AD N(%)	AMEs occurred, but no AD N(%)	No AMEs occurred but AD occurred, N(%)	AMEs occurred before AD, N (%)	Overall（N=22)**	p-value
SIPAT-J total score	<18 (median)	7 (46.7)	5 (33.3)	2 (13.3)	1 (6.7)	15 (100)	0.834
	≥18	2 (28.6)	3 (41.9)	1 (14.3)	1 (14.3)	7 (100)	
Patient's readiness level	<8 (median)	7 (58.3)	4 (33.3)	1 (8.3)	0 (0)	12 (100)	0.218
	≥8	2 (20.0)	4 (40.0)	2 (20.0)	2 (20.0)	10 (100)	
Social support system	<6 (median)	6 (42.9)	5 (35.7)	2 (14.3)	1 (7.1)	14 (100)	1.000
	≥6	3 (33.3)	3 (37.5%)	1 (12.5)	1 (12.5)	8 (100)	
Psychological stability and psychopathology	<1 (median)	7 (50.0)	4 (28.6)	2 (14.3)	1 (7.1)	14 (100)	0.724
	≥1	2 (25.0)	4 (50)	1 (12.5)	1 (12.5)	8 (100)	
Lifestyle and substance use	<3 (median)	6 (46.2)	4 (30.8)	2 (15.4)	1 (7.7)	13 (100)	0.915
	≥3	3 (33.3)	4 (44.4)	1 (11.1)	1 (11.1)	9 (100)	

## Discussion

To the best of our knowledge, this is the first study to investigate the association between AD, one of the most prevalent psychiatric disorders and any version of the SIPAT in VAD patients treated as BTT. Given that AMEs were considered a significant factor contributing to the onset of AD in BTT patients, participants were classified into four groups based on the development of AD and the occurrence of AMEs prior to AD. These four groups were further subdivided into high and low SIPAT-J score categories. Associations between these eight groups were then analyzed; however, no significant associations were found.

The most possible explanation for this result is the small sample size, which may have limited the statistical power of the analysis. Another explanation is that this research was censored at the time of heart transplantation or death, making it impossible to equalize the observation periods of each patient. The intervals between diagnosis of disease requiring heart implantation, psychiatric evaluation and VAD implantation also varied among patients. Psychosocial findings may have changed in those who had longer intervals between psychosocial evaluation and the censored time.

Also, the number of patients with AD alone may have been underestimated in the present study. No published studies have assessed the prevalence of specific psychiatric disorders in large numbers of VAD patients; however, some studies have reported varying rates of AD from 37-66% [12]. The slightly low prevalence (35.7%) in the present study may be because AD was identified only in those followed up as having delirium and/or insomnia. Also, all patients in this study were hospitalized and did not leave the hospital after the VAD implantation surgery. Therefore, they were in the 'implantation hospitalization' phase, as pointed out by Abisire et al. [13] and Okam et al. [14]. This phase is characterized by recovery from surgery and the acquisition of basic VAD skills. It is possible that a longer period of follow-up might consequence in an increased number of patients with AD.

Moreover, SIPAT is a measure originally designed to assess organ transplantation outcomes. Therefore, other psychosocial or environmental factors the SIPAT does not evaluate may be relevant in predicting outcomes for patients undergoing LVAD implantation [15]. Indeed, the stressors experienced by the participants who developed AD in this study were changes in life created by VAD, such as family dysfunction and loss of future perspective, which had occurred because of the device. These factors are not included in SIPAT. This finding supports previous research claiming that life with VAD does not meet the pre-implantation expectations of many patients [16,17]. Therefore, if the psychosocial assessment for VAD implantation candidates included how the patient and their family envision their life after VAD implantation in detail, those at risk of developing AD may be predictable.

We considered SIPAT to be a predictor of AD, with AME specifically influencing the onset of AD. However, studies on the association between SIPAT and AME remain controversial. Bui et al. reported an association between higher SIPAT scores and major bleeding [9], while Cagliostro et al. found no association with VAD-related complications or mortality [10]. Sperry et al. found that SIPAT scores were associated with cumulative adverse cardiac events but not with mortality or time to the first adverse event [18]. More recent research has examined specific SIPAT domains, showing partial associations with AMEs. For example, a study conducted by Olt et al. found that patient readiness was associated with noncardiac and cardiac readmissions, social support was associated with device-related readmissions and psychological stability, and psychopathology was associated with hemocompatibility and cardiac readmissions, as well as mortality [19]. Chesher et al. found that the patient readiness level score was significantly associated with mortality at the two-year mark [15]. These findings suggest that SIPAT may not fully capture the psychosocial and clinical factors affecting outcomes in VAD patients treated as BTT. While it is useful for transplant candidates, SIPAT may miss VAD-specific challenges.

An additional finding was that more than half of the patients referred to the psychiatric team in this study had psychiatric diagnoses, with delirium and/or insomnia being the most common. Cognitive dysfunction, including delirium and sleep disturbances, is frequently observed in postsurgical patients [20], as confirmed by this study’s findings.

This preliminary and retrospective study had several limitations. First, as mentioned previously, it was a single-center design with a small sample size. Furthermore, it was unclear if AD stemmed from preexisting psychosocial vulnerability or from brain vulnerability due to post-VAD delirium. The absence of a control group, such as patients receiving DT, further complicates interpretation within the BTT context. Despite these limitations, this study marks an important step in identifying factors relevant to psychosocial risk, especially for AD, among VAD patients awaiting transplantation.

## Conclusions

This study investigated the association between AD and SIPAT-J scores in VAD patients treated as BTT, providing novel insights into the psychosocial dimensions that may influence these patients' experiences. While no significant differences in SIPAT-J scores were observed across patient groups, our findings suggest that SIPAT may inadequately capture the unique stressors VAD patients face, particularly those that emerge post VAD implantation and impact family dynamics and future prospects. These findings underscore the importance of developing a more tailored approach to psychosocial assessment for VAD implantation candidates, with an emphasis on understanding patient and family expectations of life with a VAD. Despite the limitations of this retrospective, single-center study, our findings contribute to a growing understanding of the complexities in psychosocial assessment and psychiatric vulnerability in VAD patients. 
